# In vitro antibacterial and antifungal activities of extracts and fractions of leaves of *Ricinus communis* Linn against selected pathogens

**DOI:** 10.1002/vms3.772

**Published:** 2022-02-19

**Authors:** Bedaso Kebede, Workineh Shibeshi

**Affiliations:** ^1^ Department of Animal Products Veterinary Drug and Animal Feed Quality Assessment Centre of Ethiopian Veterinary Drug and Animal Feed Administration and Control Authority Addis Ababa Ethiopia; ^2^ Department of Pharmacology and Clinical Pharmacy School of Pharmacy College of Health Sciences Addis Ababa University Addis Ababa Ethiopia

**Keywords:** antibacterial activity, antifungal activity, MBC, MIC, *R. communis*

## Abstract

**Introduction:**

Infectious disease impacts are reduced due to the development of antimicrobial agents. However, the effectiveness of antimicrobial agents is reduced over time because of the emergence of antimicrobial resistance. To overcome these problems, scholars have been searching for alternative medicines. *Ricinus communis* is used as a traditional treatment for bovine mastitis, wound infection, and other medicinal purposes.

**Objective:**

The objective of the present study was to further evaluate the antimicrobial activities of *R. communis* leaf extracts and fractions.

**Methods:**

*R. communis* leaves were macerated in methanol and acetone. The methanol extract showed better antimicrobial activity and subjected to further fractionation via increasing polarity of solvents (n‐hexane, chloroform, ethyl acetate, and aqueous). Test microorganisms included in the study were six laboratory reference bacteria (*Escherichia coli, Staphylococcus aureus, Streptococcus agalactiae, Kleibsella pneumoniae, Pseudomonas aeruginosa* and *Streptococcus pyogenes*), two clinical isolate bacteria (*E. coli* and *S. aureus*), and *Candida albicans*. The agar well diffusion method was employed to determine antimicrobial activity. The minimum inhibitory concentrations (MIC) and minimum bactericidal/fungicidal concentrations (MBC/MFC) were determined through broth microdilution.

**Results:**

The results indicated that the best antimicrobial activity for ethyl acetate fraction ranged from 14.67 mm (clinical *E. coli*) to 20.33 mm (*S. aureus*) at 400 mg/ml, however, n‐hexane exhibited the lowest antimicrobial activity. Among the tested fractions, ethyl acetate fraction showed the lowest MIC values ranged from 1.5625 mg/ml (*S. aureus*) to 16.67 mg/ml (*Candida albicans*). The ethyl acetate fraction showed bactericidal activity against all tested microorganisms.

**Conclusion:**

Hence, ethyl acetate fraction of crude methanol extract exhibited the best antimicrobial activity.

## Graphical abstract

Antimicrobial activity of *Ricinus communis* leaf extract

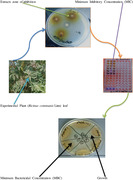

## 1 INTRODUCTION

Infectious diseases are exacerbated due to the existence of zoonotic diseases and antimicrobial resistance (Rwego et al., 2008; Uchil et al., 2014). Hence, several surveillances have been conducted on antimicrobial resistance in different countries that indicated the development of drug resistance by different pathogens to the same or different drugs are increasing from time to time with variations from region to region (Berhe et al., 2021; Brzychczy‐Wock et al., 2013; Mshana et al., 2013; Ventola, 2015). The augmentations of antimicrobial resistances have harmed both human and animal health, exposing to longer periods of hospitalisation and affecting treatment costs (Bedasa et al., 2018; Getahun et al., 2008; Kerro & Tareke, 2003). Alternative medicines have been screened from a variety of plants for their pharmacological potential as secondary metabolites are less in drug adverse effects, resistance and residues (Felhi et al., 2017; Helander et al., 1998; Puupponen‐Pimia et al., 2001; Zgurskaya et al., 2015). The examples of modern drugs that come from medicinal plant studies are vinblastine, artemisinin, topotecan, teniposide, anisodamine, 3‐n‐butylphthalide, indirubin, huperzine, acetyldigoxin, theobromide, physostigmine, digitoxin and ephedrine (Helmenstine, 2021; Kong et al., 2003; Tu, 2016; Zhang, 2002).

*Ricinus communis* Linn taxonomically belongs to the family of Euphorbiaceae and it is a sole species in the monotypic genus *Ricinus*. The vernacular names are ‘Qobboo’ (in Afan Oromo), ‘Castor oil plant or castor bean’ (in English), and ‘Gulo’ (in Amharic). It grows in altitude ranging from 400 to 4500 m above sea level in tropics and temperate regions of the world. The plant grows perennially as high as 5–10 m with a 15 cm thick and hollow trunk and leaves. It has a green or reddish colour, alternate, stipulate, long petiolate and a membranous lobe of a leaf and fruit with a thorny capsule covering a seed. It has been reproduced with mixed pollination of self‐pollination (geitonogamy) and out‐crossers by wind pollination (anemophily) or insect pollination (or entomophily) (Edwards et al., 1995; Neelam & Singh, 2015).

*R. communis* in Ethiopia is used in the treatment of blackleg and actinomycosis (Bayecha et al., 2018), diarrhoea, wound and skin rashes/dermatitis (Gijan & Dalle, 2019; Mengesha & Dessie, 2018) and bovine mastitis (Romha et al., 2015). The studies on validation of antimicrobial activity of *R. communis* leaf extracts were conducted using different solvents in Pakistan and Ghana, and methanol extract was reported to have a promising antimicrobial potential (Naz & Bano, 2012; Suurbaar et al., 2017). According to a study report on antibacterial activity of *R. communis* leaf in Ethiopia, organic solvent extracts exhibited better activity than the aqueous one (Abew et al., 2014). However, methanol was not used for extraction in Abew et al. (2014) and none has been done on the antimicrobial activities of solvent fractions of *R. communis* leaf.

Antimicrobial activities of medicinal plants are not only determined by plant species. There are also other factors such as altitude, temperature, illumination and moisture. These factors have regulated accumulation and metabolism of secondary metabolites in medicinal plants. Additionally, differences in the location of medicinal plants have contributed to the presence of different active ingredients and their concentrations (Liu et al., 2016). The test pathogens were selected based on their ability of causing a variety of diseases in humans and animals and the traditional claims on usage of *R. communis* leaf as ethnomedicine in the country. Therefore, the current study was intended to compare antimicrobial activities of methanol and acetone extracts, evaluate antimicrobial activities of solvent fractions of the best performed crude extract among the two extracts and characterise phytochemical constituents of the solvent fractions.

## 2 METHODS

### 2.1 Plant authentication and collection

The experimental plant was verified based on the works of Edwards et al. (1995) on the description of flora of Ethiopia and Eritrea before collection at the field and then authenticated by a Plant Taxonomist, Mr. Melaku Wondafrash, at the national herbarium of the College of Natural and Computational Sciences, Addis Ababa University. The plant was collected from the Sululta district, Finfinne city surrounding special zone, Oromia regional state, Ethiopia which is located at about 25 km from the capital city in October, 2019.

### 2.2 Extraction of the plant

The extraction was performed according to Ogbiko et al. (2018). The powdered leaves of 200 g were weighed on an analytical balance (Mettler Toledo, Switzerland) and macerated in 1000 ml of absolute methanol and acetone in Erlenmeyer flask at the ratio of 1:5 and after 3 days, the extract was collected and re‐macerated. Collection of the extract was carried out at interval of 3 days, so leaves were macerated totally for 9 days with the intermittent shaking on the rotary‐shaker (VWR DS‐500; The Lab World Group, Boston, MA, USA). The pool of collected extracts was first filtered through sieve mesh then followed by filtration via Whatman no. 1 by using filtration apparatus or unit. A filtrate of extracts was concentrated in a rotary evaporator (Buchii model R‐200, Switzerland) at 40°C temperature and 40 revolutions per minute (RPM) until solvents were completely removed and solid extracts were formed.

### 2.3 Crude extract solvent fractionation

The crude methanol extract was subjected to further solvent fractionation by increasing polarity including n‐hexane, chloroform, ethyl acetate and aqueous. Voukeng et al.’s (2017) method was used for solvent fractionation with modification on the concentration of extract residue between fractionation intervals. The methanol extract was not completely dissolved in water so 90% methanol solvent was used instead of it. The methanol extract (60 g) was weighed on an analytical balance and subjected to dissolve completely in 100 ml of 90% methanol (10 ml water and 90 ml methanol) in the beaker. The completely dissolved 100 ml methanol extract was mixed with 100 ml n‐hexane for solvent partitioning in a separatory funnel having a capacity of 250 ml. The separatory funnel‐contained mixture was fixed to the standing stage pole and waited until a clear and separated layer formed between the two solvents. Once a clear layer formed, the methanol part was taken first carefully to a beaker and n‐hexane partition to another container. This procedure was repeated three times and the n‐hexane partition was collected together for future concentration. The remaining methanol extract solution was subjected to evaporation in a rotary evaporator at 40°C and 40 rpm to remove methanol solvent. Then, 90 ml of water was added to the concentrated methanol extract to form a 100 ml aqueous solution. The 100 ml aqueous solution of methanol extract was mixed with 100 ml of chloroform in the separatory funnel. The separatory funnel was fixed on the standing stage pole and waited until a clear layer formed between the aqueous solution of methanol extract and chloroform. The chloroform portion was held at the lower layer and collected first in the container and the aqueous portion in another container. It was replicated three times and the chloroform portion pooled in the container for later concentration. The remnant aqueous portion of methanol extract was concentrated on a rotary evaporator to remove the remaining chloroform. The concentrated 100 ml aqueous portion of methanol extract was mixed with 100 ml ethyl acetate in the separatory funnel. The separatory funnel was fixed on the standing stage pole and waited till a clear layer appeared between aqueous fraction and ethyl acetate fraction. It was repeated three times, and the aqueous fraction and ethyl acetate fraction were collected in different containers. The aqueous fraction was lyophilised by a lyophiliser (Operon, Korea vacuum limited, Korea), but n‐hexane, chloroform and ethyl acetate fractions were concentrated in a rotary evaporator.

### 2.4 Preliminary phytochemical screening

The screening of the phytochemical constituents such as alkaloids, flavonoids, terpenoids, phenols, tannins, steroids, saponins, anthraquinones and cardiac glycosides was performed using different chemicals and reagents for the detection of secondary metabolites in extracts and fractions (Ayoola et al., 2008; Nwadiaro et al., 2015; Shetty et al., 2016; Santhi & Sengottuvel, 2016).

### 2.5 Test organisms

Microorganisms selected for the experiment were standard strains including *Staphylococcus aureus* (ATCC 25923), *Streptococcus agalactiae* (ATCC 12386), *Streptococcus pyogenes* (ATCC 19615), *Escherichia coli* (ATCC 25922), *Pseudomonas aeruginosa* (ATCC 27853) and *Klebsiella* pneumoniae (ATCC 700603) brought from Ethiopian Public Health Institute, and clinical isolates of *Staphylococcus aureus* and *Escherichia coli* obtained from Animal Products, Veterinary Drug and Animal Feed Quality Assessment Centre of Veterinary Drug and Animal Feed Administration and Control Authority, and *Candida albicans* (ATCC10231) brought from Ethiopian Biodiversity Institute. The Gram staining, selective media, haemolysin and catalase test were conducted to confirm test microorganisms according to CLSI (2008) and Brown and Lowbury (1965).

### 2.6 Standard drugs

Gentamicin 10 μg disc was used as positive control against bacteria and brought from Animal Products, Veterinary Drug, and Animal Feed Quality Assessment Centre of Ethiopian Veterinary Drug and Animal Feed Administration and Control Authority. Amphotericin‐B (20 μg/ml) was used as a positive control against fungus and obtained from the Ethiopia Food and Drug Administration and Control Authority.

### 2.7 Antibacterial activity

The brain heart infusion (BHI) broth was prepared for streptococcal species and nutrient broth for other test bacteria. Overnight cultured 3–5 distinct colonies of bacteria based on their colony size were inoculated into 4 ml broth media and incubated at 37°C overnight. The nutrient or BHI broth was added to the overnight incubated bacterial suspension and vortexed on a vortex mixer (Fisher Scientific Ltd., England) for 1 min to attain uniform distribution. The vortexed bacterial suspension was adjusted to 0.5 McFarland standards (Remel, Lenexa Kansas 66215, USA) (equivalent to 1–2 × 10^8^ CFU/ml) through contrasting against white paper black line striped and was used for experiment within 15 min (CLSI, 2015).

The 100 μl of adjusted bacterial suspension was pipetted using a micropipette and applied on the surface of Mueller Hinton agar and was swabbed at 60^o^ rotation to uniformly distribute bacteria throughout media surface using a cotton swab. The swabbed Mueller Hinton agar stood for 15 min to provide time for the attachment of bacteria on the media. After that, the sterilised cork borer of 6 mm diameter was perforated with the swabbed media to create 6 mm diameter wells. At the time of punching media for different test bacteria, the cork borer was sterilised by immersing in alcohol and burning with Bunsen burner flames (Gonelimali et al., 2018; Umer et al., 2013). The concentration of extracts for the experiment was determined based on a previous study on the plant (Abew et al., 2014). The created wells were filled with 50 μl extracts or fractions at a concentration of 400, 200 and 100 mg/ml, and negative control, but the positive control disc (gentamicin) was placed on the media surface. After all the wells on the Petri dishes were filled, and the positive control was placed on Petri dishes, then the Petri dishes were placed in the refrigerator at 4°C for 2 h to facilitate diffusion of extracts or fractions in the media. Subsequently, Petri dishes were incubated at 37°C for 24 h in the incubator (BioTechnics India). The inhibition zone diameter after 24 h incubation was measured by a ruler in millimetre and recorded (Abew et al., 2014; Ohikhena et al., 2017; Suurbaar et al., 2017). The experiment was done in triplicate.

### 2.8 Antifungal activity

The *Candida albicans* was cultured on sabouraud dextrose agar and incubated overnight. The overnight incubated yeast culture was inoculated into normal saline (0.85%). The inoculated normal saline was vortexed on a vortex mixer and adjusted to 0.5 McFarland standards (equivalent to 1–5 × 10^6^ cells/ml) by contrasting against white paper black line striped (EUCAST, 2003). The 100 μl adjusted *Candida albicans* suspension was pipetted using a micropipette and applied on the surface of sabouraud dextrose agar and swabbed at 60^o^ rotation to uniformly distribute yeast throughout the media surface using a cotton swab. The swabbed sabouraud dextrose agar stood for 15 min to provide time for the attachment of yeast on the media. After that, the sterilised 6 mm diameter cork borer was used to perforate the swabbed media to create a 6 mm diameter of wells (Ohikhena et al., 2017). The concentration of extracts for the experiment was determined based on a previous study on the plant (Suurbaar et al., 2017). The created wells were filled with the 50 μl extracts or fractions at 400, 200 and 100 mg/ml, negative, and positive control. The inoculated Petri dishes were placed in the refrigerator at 4°C for 2 h to facilitate diffusion of extracts or fractions in the media. Next to that, Petri dishes were incubated at 37°C for 24 h in the incubator. The inhibition zone diameter after 24 h incubation was measured by a ruler in millimetre and recorded (Abew et al., 2014; Ohikhena et al., 2017; Suurbaar et al., 2017). The experiment was done in triplicate.

### 2.9 Determination of minimum inhibitory concentration (MIC)

Minimum inhibitory concentration is the minimum concentration of extracts or fractions which have inhibited the growth of microorganisms. The minimum inhibitory concentrations were determined using the broth microdilution technique for extracts or solvent fractions as their inhibition zones equal to or greater than 7 mm in agar well diffusion techniques (Taye et al., 2011).

### 2.10 Determination of minimum inhibitory concentration for pathogenic bacteria

The overnight cultured 3–5 distinct bacterial colonies were inoculated into 4 ml Mueller Hinton broth and incubated at 37°C overnight. Overnight incubated bacterial suspension that had been adjusted (0.5 McFarland standards) was diluted at a ratio of 1:20 with Mueller Hinton broth (0.5 ml bacterial suspension was added to 9.5 ml broth) and vortexed to have uniformly distributed bacterial suspension (5 × 10^6^ CFU/ml). The UV radiated sterile microtitre plate (Greiner Bio‐One, Germany) wells were filled with 100 μl Mueller Hinton broth which commenced from well 1 to 12. The serial double dilution technique was employed for extracts and fractions in broth filled wells. The serial double dilution was performed as 100 μl extracts or fractions were added to the first well and thoroughly mixed for five times by rinsing using micropipette and 100 μl of the mixture was transferred to the second well using a new micropipette tip and thoroughly mixed as above. A 100 μl of the second well mixture was pipetted using a new micropipette tip and transferred to the third well, and then thoroughly mixed as above. The process was continued until the tenth well and 100 μl mixture of the tenth well was pipetted and discarded to have an equal volume of fluid in wells (CLSI, 2015).The twofold serially diluted concentrations of extracts for the experiment were determined from a previous study on the plant. The serially diluted concentrations used in the experiment were 200, 100, 50, 25, 12.5, 6.25, 3.125, 1.5625, 0.78125 and 0.3906 mg/ml (Abew et al., 2014). The 100 μl broth‐filled 11th and 20th wells were used as growth and sterility control, respectively. The 10 μl diluted bacterial suspension (10% of 100 μl well volume) was pipetted to wells from eleventh to first wells to reduce contamination to sterility control and attained a final concentration of 5 × 10^5^ CFU/ml bacteria in each well, but 10 μl broth was pipetted to the 12th well. Finally, microtitre plates were sealed using parafilm and incubated at 37°C for 24 h (CLSI, 2015). The incubated microtitre plate wells were filled with 0.01% resazurin sodium salt indicator from 12th to 1st well and incubated for 2 h at 37°C. The resazurin sodium salt reaction with actively growing microorganisms produces colour changes which are important to determine the MIC of extracts or fractions based on colour changes. The blue or purple colour appears if the growth of microorganisms is inhibited, while pink or colourless change is observed for those actively growing cells which reduced resazurin sodium salt to resorufin. Resazurin sodium salt solution was prepared by dissolving 0.01 g in 100 ml sterile distilled water and filtered through a 0.2 μm pore size filter paper and stored in a dark container at 4°C refrigerator until use (Blazic et al., 2019; Ohikhena et al., 2017). The experiment was performed in triplicate.

### 2.11 Determination of minimum inhibitory concentration for pathogenic fungi

Overnight cultured colonies of yeast were inoculated into sabouraud dextrose broth and incubated at 37°C overnight. Overnight incubated yeast suspension which had been adjusted (0.5McFarland standard) was diluted at a ratio of 1:20 with sabouraud dextrose broth (0.5 ml yeast suspension was added to 9.5 ml broth) and vortexed to have uniformly distributed yeast suspension (0.5–2.5 × 10^5^ CFU/ml). The sterile microtitre plate wells were filled with 100 μl broth of sabouraud dextrose from well one to twelve. The serial double dilution technique was employed for extracts and fractions in broth filled wells commenced from the first to tenth wells. The serial double dilution was performed as 100 μl extracts or fractions were added to the first well and thoroughly mixed five times by rinsing using a micropipette and 100 μl of the mixture was transferred to the second well using a new micropipette tip and thoroughly mixed as above. A 100 μl of the second well mixture was pipetted using a new micropipette tip and transferred to the third well and thoroughly mixed as above. The process was continued until the tenth well and 100 μl mixture of the tenth well was pipetted and discarded to have an equal volume of fluid in the wells (EUCAST, 2003). The twofold serially diluted concentrations of extracts for the experiment were determined from a previous study on the plant. The serial double dilution concentrations used in the experiment were 200, 100, 50, 25, 12.5, 6.25, 3.125, 1.5625, 0.78125 and 0.3906 mg/ml (Suurbaar et al., 2017). The 100 μl broth‐filled 11th and 20th wells were used as growth and sterility control, respectively. The 10 μl diluted yeast suspension (10% of 100 μl broth volume) was pipetted to wells from the eleventh to first wells to reduce contamination on sterility control and the attained final concentration of yeast suspension (2.5 × 10^4^ CFU/ml) in each well, but 10 μl broth was pipetted to the 12th well. The filled microtitre plate wells were sealed by parafilm and incubated at 37°C for 24 h (CLSI, 2015; EUCAST, 2003). The incubated microtitre plate wells were filled with 0.01% resazurin sodium salt indicator from the 12th to the 1st well and incubated for 2 h at 37°C. The MIC of extracts and fractions were determined as blue or purple resazurin colour changed to pink or colourless (Blazic et al., 2019; Ohikhena et al., 2017). The experiment was done in triplicate.

### 2.12 Determination of minimum bactericidal concentration (MBC)

The minimum bactericidal concentration was determined through sub‐culturing of 10 μl content of microtitre plate well which is greater or equal to the lowest minimum inhibitory concentration on the Mueller Hinton agar and incubated for 24 h. After 24 h incubation, the Petri dish was assessed for the presence of growth, and the minimum concentration of extracts or fractions with no visible growth was taken as a minimum bactericidal concentration (Akinduti et al., 2019). The experiment was done in triplicate.

### 2.13 Determination of minimum fungicidal concentration (MFC)

The minimum fungicidal concentration was determined through sub‐culturing of 10 μl content of microtitre plate well which is greater or equal to the lowest minimum inhibitory concentration on the sabouraud dextrose agar and incubated for 24 h. After 24 h incubation, the Petri dish was assessed for the presence of growth, and the minimum concentration of extracts or fractions with no visible growth was taken as minimum fungicidal concentration (Akinduti et al., 2019). The experiment was done in triplicate.

### 2.14 Data analysis

The data were entered into an excel spreadsheet for statistical analysis using Statistical Package for Social Science (SPSS) version 20. The descriptive statistics, one‐way ANOVA, Tukey's post hoc test and linear regression *R* ^2^ (Coefficient of determination) were utilised for statistical analysis and inference. The descriptive statistics were employed for calculation of group mean of inhibition zone diameter as mean ± SEM. The one‐way ANOVA was performed to determine the significant difference among group means. Whereas, Tukey's post hoc test followed one‐way ANOVA to determine the significant difference between each group mean. The linear regression *R* ^2^ was calculated to determine the concentration dependence of extracts and fractions on antimicrobial activities against test microorganisms. Statistically significant differences were declared at a *p* value of less than 0.05.

## 3 RESULTS

### 3.1 Antibacterial activity

#### 3.1.1 Agar well diffusion assay

The inhibition zone diameter was observed for extracts, fractions and positive control, but not for the negative control. The highest inhibition zone diameter of the methanol extract determined against Gram‐positive bacteria was 17.33 mm against *S. pyogenes* and Gram‐negative bacteria was 14.67 mm against *P. aeruginosa*. However, the highest concentration of methanol extract against *K. pneumoniae* produced the lowest inhibition zone diameter of 12.67 mm. The highest inhibition zone diameter of acetone extract observed against Gram‐positive bacteria was 14.33 mm against *S. pyogenes* and Gram‐negative bacteria was 13.33 mm against *E. coli*. Nevertheless, the highest concentration of acetone extract against *K*. pneumonia produced the lowest inhibition zone diameter of 11.67 mm (Tables 1 and 2). The highest inhibition zone diameter of ethyl acetate fraction observed against Gram‐positive was 20.33 mm against *S. aureus* and Gram‐negative bacteria was 16.67 mm against *P. aeruginosa*. But, n‐hexane fraction produced the lowest inhibition zone diameter and no antibacterial activity against *P. aeruginosa*. Additionally, ethyl acetate fraction produced the highest inhibition zone diameter than the crude extract of methanol, acetone, and other solvent fractions (Tables 1, 2, 3, 4). The highest inhibition zone diameter of the ethyl acetate fraction observed against clinical isolate of Gram‐positive bacteria was 17.67 mm against *S. aureus* and Gram‐negative was 14.67 mm against *E. coli* (Table 5).

**TABLE 1:** Mean inhibition zone diameter (mm) of *R. communis* leaf crude extract against Gram‐positive bacteria

		*S. aureus*		*S. agalactiae*		*S. pyogenes*	
Crude extract		Mean ± SEM	*R* ^2^	Mean ± SEM	*R* ^2^	Mean ± SEM	*R* ^2^
Methanol	100 mg/ml	10.67 ± 0.333^a3c1d3^	0.876	10.33 ± 0.333^a3d3^	0.893	12.67 ± 0.333^a3c2d3^	0.831
	200 mg/ml	12.67 ± 0.333^a3b1d1^		12.00 ± 0.577^a3d2^		15.67 ± 0.333^b2d1^	
	400 mg/ml	15.00 ± 0.577^a3b3c1^		14.67 ± 0.333^a1b3c2^		17.33 ± 0.333^b3c1^	
Acetone	100 mg/ml	9.67 ± 0.333^a3c1d3^	0.916	9.67 ± 0.333^a3c1d2^	0.809	9.67 ± 0.333^a3b1d3^	0.932
	200 mg/ml	11.33 ± 0.333^a3b1d2^		12.00 ± 0.577^a3b1^		11.67 ± 0.333^a3b1d2^	
	400 mg/ml	13.67 ± 0.333^a3b3c2^		13.67 ± 0.333^a2b2^		14.33 ± 0.333^a2b3c2^	
Gentamicin	10 μg	20.67 ± 0.333		17.00 ± 0.577		16.67 ± 0.333	

Values expressed as mean ± SEM for *n* = 3. The mean comparisons for different extracts and Gentamicin 10 μg (control) were performed by one‐way ANOVA followed by Tukey's HSD post hoc multiple comparison test. Where, compared to ^a^positive control, ^b^100 mg/ml, ^c^200 mg/ml and ^d^400 mg/ml. ^1^ *p* < 0.05, ^2^ *p* < 0.01, ^3^ *p* < 0.001. *R* ^2^ = coefficient of determination.

**TABLE 2:** Mean inhibition zone diameter (mm) of *R. communis* leaf crude extract against Gram‐negative bacteria

		*E. coli*		*K. pneumoniae*		*P. aeruginosa*	
Crude extract		Mean ± SEM	*R* ^2^	Mean ± SEM	*R* ^2^	Mean ± SEM	*R* ^2^
Methanol	100 mg/ml	10.67 ± 0.333^a3d2^	0.790	9.67 ± 0.333^d2^	0.875	11.33 ± 0.333^a3d3^	0.889
	200 mg/ml	12.00 ± 0.577^a3^		10.67 ± 0.333^d1^		12.67 ± 0.333^a3d1^	
	400 mg/ml	13.67 ± 0.333^a3b2^		12.67 ± 0.333^a2b2c1^		14.67 ± 0.333^a1b3c1^	
Acetone	100 mg/ml	9.67 ± 0.333^a3c1d3^	0.893	8.33 ± 0.333^c1d2^	0.813	9.67 ± 0.333^a3d2^	0.843
	200 mg/ml	11.33 ± 0.333^a3b1d1^		10.33 ± 0.333^b1^		10.67 ± 0.333^a3d1^	
	400 mg/ml	13.33 ± 0.333^a3b3c1^		11.67 ± 0.333^b2^		12.33 ± 0.333^a3b2c1^	
Gentamicin	10 μg	19.33 ± 0.333		10.00 ± 0.577		16.33 ± 0.333	

Values expressed as mean ± SEM for *n* = 3. The mean comparisons for different extracts and Gentamicin 10 μg (control) were performed by one‐way ANOVA followed by Tukey's HSD post hoc multiple comparison test. Where, compared to ^a^positive control, ^b^100 mg/ml, ^c^200 mg/ml and ^d^400 mg/ml. ^1^ *p* < 0.05, ^2^ *p* < 0.01, ^3^ *p* < 0.001. *R* ^2^ = coefficient of determination.

**TABLE 3:** Mean inhibition zone diameter (mm) of *R. communis* leaf solvent fractions of methanol extract against Gram‐positive bacteria

		*S. aureus*		*S. agalactiae*		*S. pyogenes*	
Solvent fraction		Mean ± SEM	*R* ^2^	Mean ± SEM	*R* ^2^	Mean ± SEM	*R* ^2^
n‐Hexane	100 mg/ml	7.33 ± 0.333^a3d2^	0.746	7.33 ± 0.333^a3d2^	0.764	7.33 ± 0.333^a3d1^	0.723
	200 mg/ml	8.67 ± 0.333^a3^		8.33 ± 0.333^a3^		8.33 ± 0.333^a3^	
	400 mg/ml	9.67 ± 0.333^a3b2^		10.00 ± 0.577^a3b2^		9.33 ± 0.333^a3b1^	
Chloroform	100 mg/ml	7.33 ± 0.333^a3c1d2^	0.750	7.67 ± 0.333^a3c1d2^	0.754	7.33 ± 0.333^a3c2d3^	0.750
	200 mg/ml	9.67 ± 0.333^a3b1^		9.67 ± 0.333^a3b1^		9.67 ± 0.333^a3b2^	
	400 mg/ml	10.67 ± 0.333^a3b2^		10.67 ± 0.333^a3b2^		10.67 ± 0.333^a3b3^	
Ethyl acetate	100 mg/ml	15.33 ± 0.333^a3c2d3^	0.928	12.67 ± 0.333^a3c1d3^	0.898	13.33 ± 0.333^a2c2d3^	0.864
	200 mg/ml	17.67 ± 0.333^a2b2d2^		15.00 ± 0.577^a1b1d2^		16.33 ± 0.333^b3c1^	
	400 mg/ml	20.33 ± 0.333^b3c2^		17.67 ± 0.333^b3c2^		18.33 ± 0.333^b3c1^	
Aqueous	100 mg/ml	12.67 ± 0.333^a3d2^	0.893	10.67 ± 0.333^a3c1d3^	0.893	13.67 ± 0.333^a3c1d3^	0.916
	200 mg/ml	14.33 ± 0.333^a3d1^		12.33 ± 0.333^a3b1d1^		15.33 ± 0.333^a1b1d1^	
	400 mg/ml	16.33 ± 0.333^a2b2d1^		14.33 ± 0.333^a2b3c1^		17.33 ± 0.333^b3c1^	
Gentamicin	10 μg	20.67 ± 0.667		17.33 ± 0.333		17.33 ± 0.333	

Values expressed as mean ± SEM for *n* = 3. The mean comparisons for different crude methanol extract's solvent fractions and Gentamicin 10 μg (control) were performed by one‐way ANOVA followed by Tukey's HSD post hoc multiple comparison test. Where, compared to ^a^positive control, ^b^100 mg/ml, ^c^200 mg/ml and ^d^400 mg/ml. ^1^ *p* < 0.05, ^2^ *p* < 0.01, ^3^ *p* < 0.001. *R* ^2^ = coefficient of determination.

**TABLE 4:** Mean inhibition zone diameter (mm) of *R. communis* leaf solvent fractions of methanol extract against Gram‐negative bacteria

		*E. coli*		*K. pneumoniae*		*P. aeruginosa*	
Solvent fraction		Mean ± SEM	*R* ^2^	Mean ± SEM	*R* ^2^	Mean ± SEM	*R* ^2^
n‐Hexane	100 mg/ml	7.33 ± 0.333^a3d2^	0.764	7.33 ± 0.333^a2d2^	0.795	–	
	200 mg/ml	8.33 ± 0.333^a3^		8.33 ± 0.333		–	
	400 mg/ml	10.00 ± 0.577^a3b2^		9.67 ± 0.333^b2^		–	
Chloroform	100 mg/ml	7.67 ± 0.333^a3c1d2^	0.816	8.67 ± 0.333^d2^	0.875	8.67 ± 0.333^a3d2^	0.875
	200 mg/ml	9.33 ± 0.333^a3b1^		9.67 ± 0.333^d1^		9.67 ± 0.333^a3d1^	
	400 mg/ml	10.67 ± 0.333^a3b2^		11.67 ± 0.333^b2c1^		11.67 ± 0.333^a3b2c1^	
Ethyl acetate	100 mg/ml	10.67 ± 0.333^a3c1d3^	0.898	10.67 ± 0.333^c1d3^	0.945	10.67 ± 0.333^a3c2d3^	0.949
	200 mg/ml	13.00 ± 0.577^a3b1d2^		12.67 ± 0.333^a2b1d2^		13.33 ± 0.333^a2b2d2^	
	400 mg/ml	15.67 ± 0.333^a2b3c2^		15.67 ± 0.333^a3b3d2^		16.67 ± 0.333^b3c2^	
Aqueous	100 mg/ml	10.33 ± 0.333^a3d2^	0.764	10.33 ± 0.333^d2^	0.795	10.67 ± 0.333^a3d2^	0.843
	200 mg/ml	11.33 ± 0.333^a3^		11.33 ± 0.333^a1^		11.67 ± 0.333^a3^	
	400 mg/ml	13.00 ± 0.577^a3b2^		12.67 ± 0.333^a2b2^		13.33 ± 0.333^a2b2^	
Gentamicin	10 μg	19.67 ± 0.333		9.67 ± 0.333		17.00 ± 0.577	

Values expressed as mean ± SEM for *n* = 3. The mean comparisons for different crude methanol extract's solvent fractions and gentamicin 10 μg (control) were performed by one‐way ANOVA followed by Tukey's HSD post hoc multiple comparison test. Where, compared to ^a^positive control, ^b^100 mg/ml, ^c^200 mg/ml and ^d^400 mg/ml. ^1^ *p* < 0.05, ^2^ *p* < 0.01, ^3^ *p* < 0.001. No activity = –, *R* ^2^ = coefficient of determination.

**TABLE 5:** Mean inhibition zone diameter (mm) of *R. communis* leaf extracts and fractions of methanol extract against clinical isolate bacteria

		Clinical *E. coli* isolate		Clinical *S. aureus* isolate	
Extract and fraction		Mean ± SEM	*R* ^2^	Mean ± SEM	*R* ^2^
Methanol	100 mg/ml	7.67 ± 0.333^a3d2^	0.875	9.33 ± 0.333^a3d2^	0.735
	200 mg/ml	8.67 ± 0.333^a3d1^		10.67 ± 0.333^a3^	
	400 mg/ml	10.67 ± 0.333^a3b2c1^		12.00 ± 0.577^a3b2^	
Acetone	100 mg/ml	7.67 ± 0.333^a3d2^	0.875	7.33 ± 0.333^a3d2^	0.860
	200 mg/ml	8.67 ± 0.333^a3d1^		8.67 ± 0.333^a3d1^	
	400 mg/ml	10.67 ± 0.333^a3b2c1^		11.00 ± 0.577^a3b2c1^	
n‐Hexane	100 mg/ml	7.33 ± 0.333^a3d1^	0.723	7.33 ± 0.333^a3d2^	0.795
	200 mg/ml	8.33 ± 0.333^a3^		8.33 ± 0.333^a3^	
	400 mg/ml	9.33 ± 0.333^a3b1^		9.67 ± 0.333^a3b2^	
Chloroform	100 mg/ml	7.33 ± 0.333^a3d1^	0.723	8.33 ± 0.333^a3d2^	
	200 mg/ml	8.33 ± 0.333^a3^		9.33 ± 0.333^a3^	
	400 mg/ml	9.33 ± 0.333^a3b1^		10.67 ± 0.333^a3b2^	
Ethyl acetate	100 mg/ml	11.33 ± 0.333^a3d2^	0.804	13.00 ± 0.577^a3c1d3^	0.842
	200 mg/ml	13.00 ± 0.577^a3^		15.67 ± 0.333^a2b2d1^	
	400 mg/ml	14.67 ± 0.333^a2b2^		17.67 ± 0.333^b3c2^	
Aqueous	100 mg/ml	7.67 ± 0.333^a3d2^	0.843	12.00 ± 0.577^a3d2^	0.766
	200 mg/ml	8.67 ± 0.333^a3d1^		14.00 ± 0.577^a3^	
	400 mg/ml	10.33 ± 0.333^a3b2d1^		15.67 ± 0.333^a2b2^	
Gentamicin	10 μg	19.67 ± 0.333		19.67 ± 0.333	

Values expressed as mean ± SEM for *n* = 3. The mean comparisons for different extracts, crude methanol extract's solvent fractions and gentamicin 10 μg (control) were performed by one‐way ANOVA followed by Tukey's HSD post hoc multiple comparison test. Where, compared to ^a^positive control, ^b^100 mg/ml, ^c^200 mg/ml and ^d^400 mg/ml. ^1^ *p* < 0.05, ^2^ *p* < 0.01, ^3^ *p* < 0.001. *R* ^2^ = coefficient of determination.

### 3.2 Antifungal activity

#### 3.2.1 Agar well diffusion assay

The assay determined inhibition zone diameter for extracts, fractions and positive control, but not for negative control. The aqueous fraction exhibited the highest inhibition zone diameter of 21 mm, but no inhibition zone diameter was observed for n‐hexane and chloroform fractions against *C. albicans* (Table 6).

**TABLE 6:** Mean inhibition zone diameter (mm) of *R. communis* leaf extracts and fractions of methanol extract against fungi

		*Candida albicans*	
Test extract and fraction		Mean ± SEM	*R* ^2^
Methanol extract	100 mg/ml	7.33 ± 0.333^a3d2^	0.735
	200 mg/ml	8.67 ± 0.333^a3^	
	400 mg/ml	10.00 ± 0.577^a3b2^	
Acetone extract	100 mg/ml	7.33 ± 0.333^a3d2^	0.746
	200 mg/ml	8.67 ± 0.333^a3^	
	400 mg/ml	9.67 ± 0.333^a3b2^	
n‐Hexane fraction	100 mg/ml	–	
	200 mg/ml	–	
	400 mg/ml	–	
Chloroform fraction	100 mg/ml	–	
	200 mg/ml	–	
	400 mg/ml	–	
Ethyl acetate fraction	100 mg/ml	11.33 ± 0.333^a3b1d3^	0.890
	200 mg/ml	13.33 ± 0.333^a3b1d1^	
	400 mg/ml	15.33 ± 0.333^a3b3c1^	
Aqueous fraction	100 mg/ml	14.67 ± 0.333^a3c1d3^	0.928
	200 mg/ml	17.00 ± 0.577^a3b1d2^	
	400 mg/ml	21.00 ± 0.577^a1b3c2^	
Amphotericin‐B	20 μg/ml	23.33 ± 0.333	

Values expressed as mean ± SEM for *n* = 3. The mean comparisons for different extracts, crude methanol extract's fractions and amphotericin‐B 20 μg/ml (control) were performed by one‐way ANOVA followed by Tukey's HSD post hoc multiple comparison test. Where, compared to ^a^positive control, ^b^100 mg/ml, ^c^200 mg/ml and ^d^400 mg/ml. ^1^ *p* < 0.05, ^2^ *p* < 0.01, ^3^ *p* < 0.001. No activity = –, *R* ^2^ = coefficient of determination.

#### 3.2.2 Determination of minimum inhibitory concentration of extracts and fractions of methanol extract against pathogenic bacteria

The minimum inhibitory concentration of the methanol extract ranged from 6.25 mg/ml (*S. aureus*) to 25 mg/ml (*E. coli*, *K. pneumoniae*, *P. aeruginosa*) and the acetone extract ranged from 8.33 mg/ml (*S. pyogenes*) to 100 mg/ml (*K. pneumoniae*). Also, the minimum inhibitory concentration of the ethyl acetate fraction ranged from 1.5625 mg/ml (*S. aureus*) to 12.5 mg/ml (*P. aeruginosa*) and for the aqueous fraction ranging from 6.25 mg/ml (*S. aureus* and *S. pyogenes*) to 66.67 mg/ml (*K. pneumoniae*) (Tables 7 and 8). The minimum inhibitory concentration for the clinical isolate bacteria ranged from 3.125 mg/ml of ethyl acetate fraction (*S. aureus*) to 100 mg/ml of n‐hexane and chloroform fractions (*S. aureus* and *E. coli*) (Table 9).

**TABLE 7:** MIC and MBC of extracts and fractions of methanol extract against Gram‐positive bacteria

Test extract and fraction	Activities	*S. aureus* Mean ± SEM (mg/ml)	*S. agalactiae* Mean ± SEM (mg/ml)	*S. pyogenes* Mean ± SEM (mg/ml)
Methanol extract	MIC	6.25 ± 0.000	12.500 ± 0.000	6.25 ± 3.1250
	MBC	100.00 ± 0.000	100.00 ± 0.000	100.00 ± 0.000
Acetone extract	MIC	18.75 ± 6.25	16.67 ± 4.167	8.33 ± 2.083
	MBC	200.00 ± 0.000	200.00 ± 0.000	200.00 ± 0.000
n‐Hexane fraction	MIC	100.00 ± 0.00	100.00 ± 0.00	100.00 ± 0.00
	MBC	ND	ND	ND
Chloroform fraction	MIC	83.33 ± 16.667	16.67 ± 4.167	16.67 ± 4.167
	MBC	ND	ND	ND
Ethyl acetate fraction	MIC	1.5625 ± 0.00	4.17 ± 1.0417	3.125 ± 0.000
	MBC	25.00 ± 0.000	25.00 ± 0.000	50.00 ± 0.000
Aqueous fraction	MIC	6.25 ± 0.00	12.50 ± 0.000	6.250 ± 0.000
	MBC	200.00 ± 0.000	200.00 ± 0.000	200.00 ± 0.000

Mean value expressed as mean ± SEM (*n* = 3). ND = not detected.

**TABLE 8:** MIC and MBC of extracts and fractions of methanol extract against Gram‐negative bacteria

Test extract and fraction	Activities	*E. coli* Mean ± SEM (mg/ml)	*K. pneumoniae* Mean ± SEM (mg/ml)	*P. aeruginosa* Mean ± SEM (mg/ml)
Methanol extract	MIC	25.00 ± 0.000	25.00 ± 0.000	25.00 ± 0.000
	MBC	ND	ND	200.00 ± 0.000
Acetone extract	MIC	66.67 ± 16.667	100.00 ± 0.000	66.67 ± 16.667
	MBC	ND	ND	ND
n‐Hexane fraction	MIC	100.00 ± 0.000	100.00 ± 0.000	NT
	MBC	ND	ND	NT
Chloroform fraction	MIC	83.33 ± 16.667	83.33 ± 16.667	50.00 ± 0.000
	MBC	ND	ND	ND
Ethyl acetate fraction	MIC	4.17 ± 1.0417	6.250 ± 0.000	12.50 ± 0.000
	MBC	200.00 ± 0.000	200.00 ± 0.000	50.00 ± 0.000
Aqueous fraction	MIC	50.00 ± 0.000	66.67 ± 16.667	25.00 ± 0.000
	MBC	ND	ND	200.00 ± 0.000

Mean value expressed as mean ± SEM (*n* = 3). ND = not detected, NT = not tested.

**TABLE 9:** MIC and MBC of extracts and fractions of methanol extract against clinical isolate bacteria

Test extract and fraction	Activities	Clinical *E. coli* isolate Mean ± SEM (mg/ml)	Clinical *S. aureus* isolate Mean ± SEM (mg/ml)
Methanol extract	MIC	25.00 ± 0.000	12.50 ± 0.000
	MBC	ND	ND
Acetone extract	MIC	25.00 ± 0.000	12.50 ± 0.000
	MBC	ND	ND
n‐Hexane fraction	MIC	100.00 ± 0.000	100.00 ± 0.000
	MBC	ND	ND
Chloroform fraction	MIC	100.00 ± 0.000	100.00 ± 0.000
	MBC	ND	ND
Ethyl acetate fraction	MIC	6.250 ± 0.000	3.125 ± 0.000
	MBC	200.00 ± 0.000	83.33 ± 16.667
Aqueous fraction	MIC	66.67 ± 16.667	25.00 ± 0.000
	MBC	ND	ND

Mean value expressed as mean ± SEM (*n* = 3). ND = not detected.

#### 3.2.3 Determination of minimum bactericidal concentration of extracts and fractions of methanol extract

The minimum bactericidal concentration (MBC) was determined as there was no visible growth observed at the lowest concentration of extracts or solvent fractions. The MBC of methanol extract ranging from 100 mg/ml (Gram‐positive bacteria) to 200 mg/ml (*P. aeruginosa*) and was not detected in *E. coli* and *K. pneumoniae*. The minimum bactericidal concentration of the acetone extract was 200 mg/ml in Gram‐positive bacteria, but not detected in the Gram‐negative bacteria. The minimum bactericidal concentrations of n‐hexane and chloroform fractions were not detected. However, minimum bactericidal concentration of the ethyl acetate fraction ranged from 25 mg/ml (*S. aureus* and *S. agalactiae*) to 200 mg/ml (*E. coli* and *K. pneumoniae*), and in the clinical isolate bacteria, from 88.33 mg/ml (*S. aureus*) to 200 mg/ml (*E. coli*) (Tables 7, 8, 9).

#### 3.2.4 Determination of minimum inhibitory and fungicidal concentration of extracts and fractions of methanol extract against *Candida albicans*

The minimum fungistatic concentration of extracts and fractions of methanol extract ranged from 3.125 mg/ml of the aqueous fraction to 66.67 mg/ml of the methanol extract against *C. albicans*. The minimum fungicidal concentration of fractions of the methanol extract ranged from 50 mg/ml of the aqueous fraction to 200 mg/ml of the ethyl acetate fraction. However, the minimum bactericidal concentrations of the methanol and acetone extracts were not detected against *C. albicans* (Table 10).

**TABLE 10:** MIC and MFC of extracts and fractions of methanol extract against fungi

Test extract and fraction	Activities	*Candida albicans* Mean ± SEM (mg/ml)
Methanol extract	MIC	66.67 ± 16.667
	MFC	ND
Acetone extract	MIC	41.67 ± 8.333
	MFC	ND
n‐Hexane fraction	MIC	ND
	MFC	ND
Chloroform fraction	MIC	ND
	MFC	ND
Ethyl acetate fraction	MIC	16.67 ± 4.167
	MFC	200.00 ± 0.000
Aqueous fraction	MIC	3.125 ± 0.000
	MFC	50.00 ± 0.000

Mean value expressed as mean ± SEM (*n* = 3). ND = not detected.

#### 3.2.5 Physical characteristics and preliminary screening of phytochemical constituents of *Ricinus communis* leaf

The physical characteristics of *Ricinus communis* leaf extracts and fractions of the methanol extract were dark green and reddish‐brown, sticky solid inconsistency and per cent of the yield ranged from 7.5% to 41.67%. The phytochemical screening detected alkaloids, flavonoids, terpenoids, tannins, cardiac glycosides, steroids, anthraquinones, saponins and phenols in the crude methanol extract and ethyl acetate fraction of *Ricinus communis* leaf (Tables 11 and 12).

**TABLE 11:** Percentage yield and physical characteristics of extracts and fractions of methanol extract of *R. communis* leaf

Test extract and fraction	Consistency	Colour	Weight of leaf powder or extracts (g)	Weight of extracts or fractions (g)	Percentage of yield
Methanol extract	Sticky solid	Dark green	200	40	20%
Acetone extract	Sticky solid	Dark green	200	15	7.5%
n‐Hexane fraction	Semisolid	Dark green	60	10	16.67%
Chloroform fraction	Solid	Dark green	60	15	25%
Ethyl acetate fraction	Sticky solid	Reddish‐brown	60	5	8.33%
Aqueous fraction	Sticky solid	Reddish brown	60	25	41.67%

**TABLE 12:** Phytochemical constituents of extracts and fractions of methanol extract of *R. communis* leaf

	Crude extract		Solvent fractions			
Secondary metabolites	Methanol	Acetone	n‐Hexane	Chloroform	Ethyl acetate	Aqueous
Flavonoids	+	+	−	+	+	+
Alkaloids	+	+	+	+	+	+
Saponins	+	−	−	+	+	+
Cardiac glycosides	+	+	−	−	+	+
Terpenoids	+	+	−	−	+	+
Tannins	+	+	+	+	+	+
Steroids	+	−	−	−	+	+
Phenols	+	+	+	+	+	+
Anthraquinones	+	−	−	−	+	−

−, absence; +, presence.

## 4 DISCUSSION

The current study aimed to investigate antimicrobial activities of extracts and fractions of methanol extract of *R. communis* leaf against pathogenic bacteria and *Candida albicans*. However, antibacterial activity had been done in a previous study from Gonder, Ethiopia, but this study did not include antifungal activity and methanol in extraction (Abew et al., 2014). Furthermore, previous findings have reported that methanol solvent extract exhibited the best antimicrobial activities from Ghana and Pakistan (Naz & Bano, 2012; Suurbaar et al., 2017). Both methanol and acetone extracts were also assessed for their antimicrobial activities to select the one which exhibited better antimicrobial activity for further solvent fractionation. There was a difference in the antimicrobial activities of the two extracts for the presence and concentration of secondary metabolites which could be affected by the type of solvent used for extraction (Liu et al., 2016).

The current study indicated that ethyl acetate fraction exhibited the highest antimicrobial activities in all tested microorganisms. Crude extracts were tested for their effects against Gram‐positive and Gram‐negative bacteria for their antimicrobial activities. Methanol extract revealed higher antimicrobial activity than acetone extract at the same concentrations. This finding agrees with that of the previous studies of Chandrasekaran and Venkatesalu (2004), Naz and Bano (2012) and Suurbaar et al. (2017). It is probably due to the capability of methanol dissolving more secondary metabolites (Chandrasekaran & Venkatesalu, 2004). Methanol and acetone extracts exhibited greater antibacterial activities against Gram‐positive bacteria than Gram‐negative bacteria in a concentration‐dependent manner. This could be because of the differences in cell surface structure between Gram‐positive and Gram‐negative bacteria. The outer membrane of Gram‐negative bacteria possesses lipopolysaccharides and lipoproteins. The lipopolysaccharides are amphipathic compounds that comprise hydrophilic polysaccharide at the core that makes up a more rigid outer membrane which slows down the diffusion of hydrophobic compounds through Gram‐negative bacteria cell membranes and acts as a barrier of permeability (Helander et al., 1998; Puupponen‐Pimia et al., 2001; Zgurskaya et al., 2015).

The solvent fractions of methanol extract exhibited antimicrobial activity in a concentration‐dependent manner except for n‐hexane fraction that showed no antibacterial activity against *P. aeruginosa*. Hexane and chloroform fractions revealed the lowest antibacterial activity and no antifungal activity. This could be due to variations in the concentration of secondary metabolites present in the solvent fractions (Osuagwu & Emi, 2013; Palmer‐Young et al., 2017). The ethyl acetate fraction revealed the highest antibacterial activity than the extracts and fractions which is in agreement with the finding of the previous study done by Voukeng et al. (2017). The aqueous fraction exhibited the highest antifungal activity followed by ethyl acetate fraction perhaps due to the capability of ethyl acetate in concentrating a greater number of secondary metabolites from partitioning of methanol extract and interaction of these phytochemical constituents. Secondary metabolites with antifungal activity are concentrated more in aqueous solvent of methanol extract (Osuagwu & Emi, 2013; Palmer‐Young et al., 2017; Sisay et al., 2019).

Means of inhibition zone diameter of extracts and fractions were significantly (*p* < 0.05) lower than the mean of inhibition zone diameter of positive control. The reason could be extracts and fractions possessed both pharmacologically active and non‐active substances whereas the control positive possessed purified and concentrated active ingredient (Ezekiel et al., 2009). However, some solvent extracts and fractions produced comparable mean of inhibition zone diameter to the positive control in the case of methanol extract and aqueous fraction against *S. pyogenes*, acetone extract and chloroform fraction against *K. pneumoniae*, and ethyl acetate fraction against all tested Gram‐positive bacteria, *P. aeruginosa* and clinical *S. aureus* at 400 mg/ml. Furthermore, the mean of inhibition zone diameter of the methanol extract, ethyl acetate fraction and aqueous fraction against *K. pneumoniae* at 400 mg/ml was significantly (*p* < 0.05) higher than the mean of inhibition zone diameter of the positive control. Mean of inhibition zone diameter of extracts and fractions against clinical isolate of *E. coli* and *S. aureus* was slightly lower than that of laboratory strains of *E. coli* and *S. aureus* which agrees with the finding of Molla et al. (2016). The resistance mechanisms such as efflux pumps, β‐lactamase production and biofilm formation could have hindered the effectiveness of antibacterial in clinical isolates than laboratory strains (Kapoor et al., 2017).

The broth microdilution technique revealed the lowest minimum inhibitory concentration for ethyl acetate fraction against pathogenic bacteria whereas the aqueous fraction was against yeast. The experiment indicated that the minimum inhibitory concentration of the broth microdilution technique was inversely proportional to the inhibition zone of the agar well diffusion technique. This is an indication of the reproducibility of an experiment (Scorzoni et al., 2007). The ethyl acetate fraction also exhibited minimum bactericidal and fungicidal concentration against all tested microorganisms. Apart from this, n‐hexane and chloroform fractions were devoid of bactericidal and fungicidal activity. This could be due to the concentration of the higher number of secondary metabolites in the ethyl acetate fraction than extract and fractions despite the detection of phytochemical constituents (Palmer‐Young et al., 2017; Sisay et al., 2019).

The maceration technique was performed for the extraction of *R. communis* leaf and yielded a higher per cent for methanol extract than acetone extract. The solvent fractionation yielded a higher per cent for aqueous fraction than the other solvent fractions. The phytochemical constituents screening revealed the presence of flavonoids, alkaloids, saponins, cardiac glycosides, terpenoids, tannins, steroids, phenols and anthraquinones in methanol extract and ethyl acetate fraction whereas anthraquinones were not detected in the aqueous fraction. Saponins, steroids and anthraquinones were absent in acetone extract, but only alkaloids, tannins and phenols were presented in n‐hexane fraction. The capacity of methanol in extracting more extract yield and phytochemical constituents is in agreement with the findings of Felhi et al. (2017) and Truong et al. (2019). The variation in types and concentration of phytochemical constituents and per cent of extract yield is because of the difference in substance solubility among solvents. The difference in solubility of a substance might be based on the physical and chemical properties of solvents and phytochemical constituents. Types, quantity and interactions of secondary metabolites present in extracts and fractions are determinants of antimicrobial activities (Cowan, 1999; Felhi et al., 2017; Palmer‐Young et al., 2017; Shafique et al., 2011).

## 5 LIMITATION OF STUDY

The current study limitation was the small number of tested microorganisms.

## 6 CONCLUSION AND RECOMMENDATIONS

The *R. communis* leaf was subjected to different solvents for extraction whereas the methanol solvent yielded more crude extract. The methanol extract contained all the screened secondary metabolites and exhibited the best antimicrobial properties against all tested microorganisms in a concentration‐dependent manner. The methanol extract exposed to different solvents for solvent fractionation indicated that the ethyl acetate fraction contained all the screened secondary metabolites and revealed the most pronounced antimicrobial activity than the extracts and other fractions, but the aqueous fraction exhibited better anticandidal activity. The current study supports the claims of use of *R. communis* leaf as traditional medicine for the treatment of infectious diseases caused by bacterial and fungal pathogens. Based on the current study findings the following points were forwarded.

- Studies should be conducted on ethyl acetate fraction to further isolate, purify, and identify bioactive principle(s) responsible for antibacterial and antifungal activities of the plant.
- Further study should be conducted on antimicrobial activities of the plant on other bacterial and fungal pathogens apart from the currently tested microorganisms.
- Mechanistic studies on isolated, purified and identified bioactive principle(s) of ethyl acetate fraction against bacterial and fungal pathogens should be conducted.
- In vivo antimicrobial study should be conducted to confirm the in vitro antimicrobial activities of the extracts and fractions of plant against the selected bacterial and fungal pathogens.

## ETHICS APPROVAL AND CONSENT TO PARTICIPATE

The study was approved by Ethics Review Board of School of Pharmacy in Addis Ababa University but no consent was needed.

## AUTHOR CONTRIBUTIONS

BK developed proposal, designed and conducted all laboratory experiments, analysed and interpreted experimental results and developed manuscript. WS participated in supervision, proposal development and manuscript preparations. Authors read and approved the final manuscript.

## COMPETING INTERESTS

The authors declare that they have no competing interests.

## CONSENT FOR PUBLICATION

Co‐authors have consented the publication of this manuscript.

### PEER REVIEW

The peer review history for this article is available at https://publons.com/publon/10.1002/vms3.772

## Data Availability

The data is available in public library of Addis Ababa University in a form of graduate student thesis.
